# Supplementary material for: Identification of photoperiod-induced specific miRNAs in the adrenal glands of Sunite sheep (Ovis aries)
Source: Front Vet Sci. 2022 Jul 22;9:888207. doi: 10.3389/fvets.2022.888207 (PMC9354845; doi:10.3389/fvets.2022.888207)
Supplement: Supplementary file 1 [file Data_Sheet_1.ZIP › Supplementary file/miRDeep2 file.zip!/miRDeep2 file/Novel_Structure/SP42A1/Novel_114.pdf]

Provisional ID	:	NC_019459.2_2897
Score total	:	3.6
Score for star read(s)	:	3.9
Score for read counts	:	-3.9
Score for mfe	:	2
Score for randfold	:	1.6
Score for cons. seed	:	
Total read count	:	4
Mature read count	:	1
Loop read count	:	0
Star read count	:	3

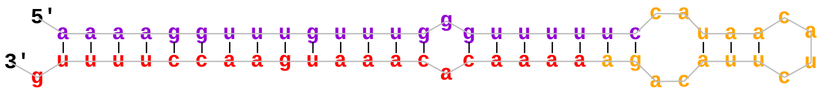

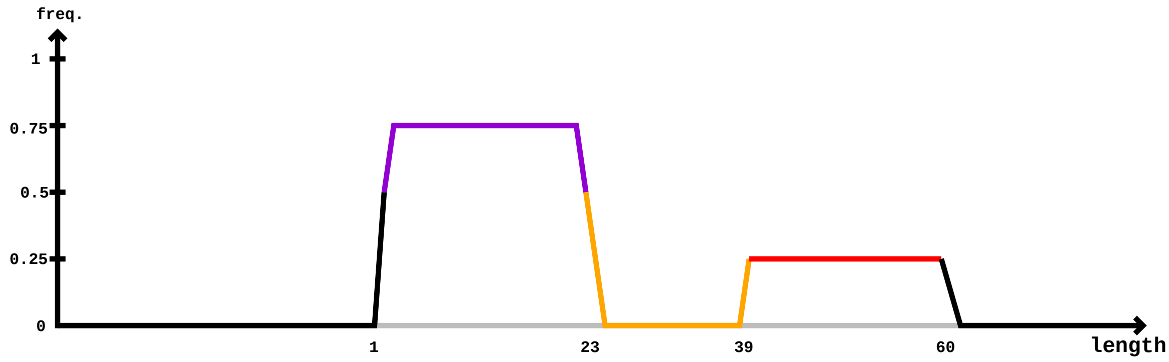

Star Mature

```
5'- cuuauacagaguaguuuuauauuggguuggccaaaagguuuguuuggguuuuuccauaacaucuuacagaaaaacacaaaugaaccuuuuggccaaucuaguauuuccaca   -3'   obs
    cuuauacagaguaguuuuauauuggguuggccaaaagguuuguuuggguuuuuccauaacaucuuacagaaaaacacaaaugaaccuuuuggccaaucuaguauuuccaca         exp
    .............((...((((((((((((((((((((((((((((.(((((((..(((....)))..))))))).))))))))))))))))))))))))))))....)).   reads   mm   sample
    ................................aaaagAuuuguuuggguuuuu..........................................................   1       1    seq
    ................................aaaagguuuguuuggguuuuuc.........................................................   1       0    seq
    .................................aaagguuuguuuggguuuuuc.........................................................   1       0    seq
    ......................................................................aaaacacaaaugaaccuuuug....................   1       0    seq
```
